# The Clinical and Histopathological Features of Cutaneous Immune-Related Adverse Events and Their Outcomes

**DOI:** 10.3390/jcm10040728

**Published:** 2021-02-12

**Authors:** Hiroki Hashimoto, Takamichi Ito, Toshio Ichiki, Yuichi Yamada, Yoshinao Oda, Masutaka Furue

**Affiliations:** 1 Department of Dermatology, Graduate School of Medical Sciences, Kyushu University, Fukuoka 812-8582, Japan; takamiti@dermatol.med.kyushu-u.ac.jp (T.I.); itoshio@dermatol.med.kyushu-u.ac.jp (T.I.); furue@dermatol.med.kyushu-u.ac.jp (M.F.); 2 Department of Anatomic Pathology, Graduate School of Medical Sciences, Kyushu University, Fukuoka 812-8582, Japan; yyamada@surgpath.med.kyushu-u.ac.jp (Y.Y.); oda@surgpath.med.kyushu-u.ac.jp (Y.O.)

**Keywords:** cutaneous immune-related adverse events, immune checkpoint inhibitor, cytotoxic T-lymphocyte associated protein 4, immunotherapy, programmed cell death 1, programmed cell death ligand 1, inflammatory eruption

## Abstract

Immune checkpoint inhibitors (ICIs) cause a variety of inflammatory eruptions. The understanding of ICI-induced inflammatory eruptions with detailed histopathological findings is not adequate, particularly in Asian populations. In this study, we retrospectively reviewed 51 patients who were histopathologically diagnosed with cutaneous immune-related adverse events (irAEs) following ICI therapy between 2014 and 2020 at the Department of Dermatology of Kyushu University Hospital. Of the 51 patients (30 men, 21 women), maculopapular rash (38/51, 74.5%), erythema multiforme (2/51, 3.9%), lichenoid reaction (3/51, 5.9%), psoriasiform reaction (3/51, 5.9%), bullous pemphigoid (3/51, 5.9%), scleroderma-like reaction (1/51, 2.0%), and Stevens–Johnson syndrome (1/51, 2.0%) were observed. The clinical and histopathological findings of these eruptions were equivalent to typical cases of common drug eruptions. The onset of maculopapular rash was relatively early (more than half of events occurred within 1 month), whereas lichenoid reactions and autoimmune diseases occurred relatively late (4–8 months). With appropriate treatment and/or interruption of ICIs, most rashes improved (50/51, 98.0%). The ICI-induced inflammatory eruptions shared similar clinical and histopathological features with classical inflammatory eruptions, but a variety of inflammatory eruptions may occur with different degrees of severity. Dermatologists play an important role in providing specialized care for cutaneous irAEs.

## 1. Introduction

Immune checkpoint inhibitors (ICIs) have emerged as key anti-tumor drugs that leverage the immune system to promote anti-tumor activity. Monoclonal antibodies directed against programmed cell death 1 (PD-1, e.g., nivolumab and pembrolizumab), programmed cell death ligand 1 (PD-L1, e.g., avelumab, atezolizumab, and durvalumab), and cytotoxic T-lymphocyte-associated protein 4 (CTLA-4, e.g., ipilimumab) have been approved for treating multiple solid tumor types, including melanoma, Merkel cell carcinoma, head and neck squamous cell carcinoma, lung cancer, urothelial carcinoma, renal cell carcinoma, and gastrointestinal cancers. Sustained anti-tumor responses can be elicited, but immune-related adverse events (irAEs) affecting multiple organs may also be triggered [1,2,3,4].

Cutaneous irAEs are the most frequent and usually the earliest irAEs arising in patients receiving ICIs. Dermatologists play an important role in evaluating and managing these cutaneous toxicities. Cutaneous irAEs include a diverse group of inflammatory eruptions. Nonspecific maculopapular rash, pruritus, and lichenoid reactions are the most prevalent subtypes [5,6,7]. Other frequent cutaneous irAEs include erythema multiforme, psoriasiform reactions, bullous pemphigoid, dermatomyositis, and oral mucosal changes [8,9]. Severe inflammatory eruptions such as Stevens–Johnson syndrome (SJS) and toxic epidermal necrolysis (TEN) have also been reported [8,9,10,11,12]. Vitiligo-like depigmentation occurs frequently in patients who receive anti-PD-1 agents for melanoma [8,9].

Clinical trials have demonstrated that cutaneous irAEs are more likely to develop during combination anti-CTLA-4 and anti-PD-1 therapy (i.e., 40.3% of patients with melanoma receiving nivolumab and ipilimumab) than during monotherapy with anti-PD-1 (25.9% of patients with melanoma receiving nivolumab) or anti-CTLA-4 (32.8% of patients with melanoma receiving ipilimumab) [13]. Previous studies have identified different rash types of irAEs and reported their characteristics, therapeutic impact, and response to treatment [5,6,14,15]. However, no report has described the clinical and histopathological findings of cutaneous irAEs in Asian populations. In this study, we analyzed cutaneous irAEs to better characterize the features of each rash.

## 2. Materials and Methods

We conducted this retrospective study in accordance with the concepts enshrined in the Declaration of Helsinki. This study was approved by the Kyushu University Institutional Ethics Committee (30-363; 27 November 2018).

We identified 51 patients who underwent skin biopsy and received a diagnosis of cutaneous irAE at the Department of Dermatology of Kyushu University Hospital between November 2014 and December 2020. The patients were referred to us for the evaluation of eruptions that developed during treatment with ICIs (nivolumab, pembrolizumab, ipilimumab, atezolizumab, avelumab, and durvalumab). Excluded patients were (1) those with inflammatory eruption attributed to another drug type other than ICIs (e.g., nonsteroidal anti-inflammatory drugs and antibiotics) and (2) those with an irAE of vitiligo alone or pruritus without inflammatory eruption. All skin biopsy samples were subjected to hematoxylin and eosin (HE) staining, and the diagnosis was confirmed by at least three experienced dermatopathologists. We extracted patients with a high possibility of cutaneous irAE based on the timing of the onset of rash, improvement after interruption of immunotherapy, histopathological findings, and the results of drug-induced lymphocyte stimulation test (DLST). Any ambiguous cases were excluded.

The medical records were reviewed and analyzed for patient demographics, underlying malignancies, and medications. The grade of rash, the duration from the start of treatment to reaction, the presence or absence of pruritus and mucosal lesions, blood eosinophil counts, and the presence or absence of other irAEs were also analyzed according to the rash type; the treatment of rash, response of rash, and impact of the rash on immunotherapy (none, temporarily interrupted, or discontinued) were analyzed according to the grade of rash. The rash grade was determined using Common Terminology Criteria for Adverse Events, version 5.0, as follows: a grade 1 rash covered <10% of the body surface area (BSA) with or without symptoms; a grade 2 rash covered 10–30% of the BSA with or without symptoms affecting instrumental activities of daily living (ADLs) or covered >30% of the BSA with or without mild symptoms without limiting self-care ADLs; a grade 3 rash covered >30% of the BSA with moderate or severe symptoms limiting self-care ADLs; and a grade 4 rash represented life-threatening consequences requiring urgent intervention including intensive care.

## 3. Results

### 3.1. Demographics, Underlying Malignancies, and Medications

Fifty-one patients with biopsy-proven cutaneous irAEs (30 men and 21 women) met the eligibility criteria. All patients were Japanese. Patient demographics, underlying malignancies, and the ICIs administered are summarized in Table 1. The most frequent cancer types were lung cancer (*n* = 18) and melanoma (*n* = 12). Other malignancies (*n* = 21) included Merkel cell carcinoma (*n* = 1), cutaneous squamous cell carcinoma of the head and neck (*n* = 6), esophageal squamous cell carcinoma (*n* = 3), gastric adenocarcinoma (*n* = 2), renal cell carcinoma (*n* = 5), urothelial carcinoma (*n* = 1), and lymphoma (*n* = 3).

Cutaneous irAEs were most commonly associated with the anti-PD-1 antibodies nivolumab (24/51, 47.1%) and pembrolizumab (13/51, 25.5%), followed by combination anti-PD-1 and anti-CTLA-4 therapy with nivolumab and ipilimumab (6/51, 11.8%), the anti-PD-L1 antibodies; atezolizumab (4/51, 7.8%); avelumab (2/51, 3.9%); and anti-CTLA-4 antibody ipilimumab (2/51, 3.9%). One patient received nivolumab in a clinical trial setting in 2014. When cutaneous irAEs occurred during the combination therapy of nivolumab and ipilimumab, the two drugs were considered to be causative even if the rash occurred during the subsequent nivolumab monotherapy (one patient). No patient received sequential therapy from ipilimumab to anti-PD-1 antibody therapy.

### 3.2. Clinical Presentations, Histopathological Diagnoses, and Grade of Rash

**Figure 1:**
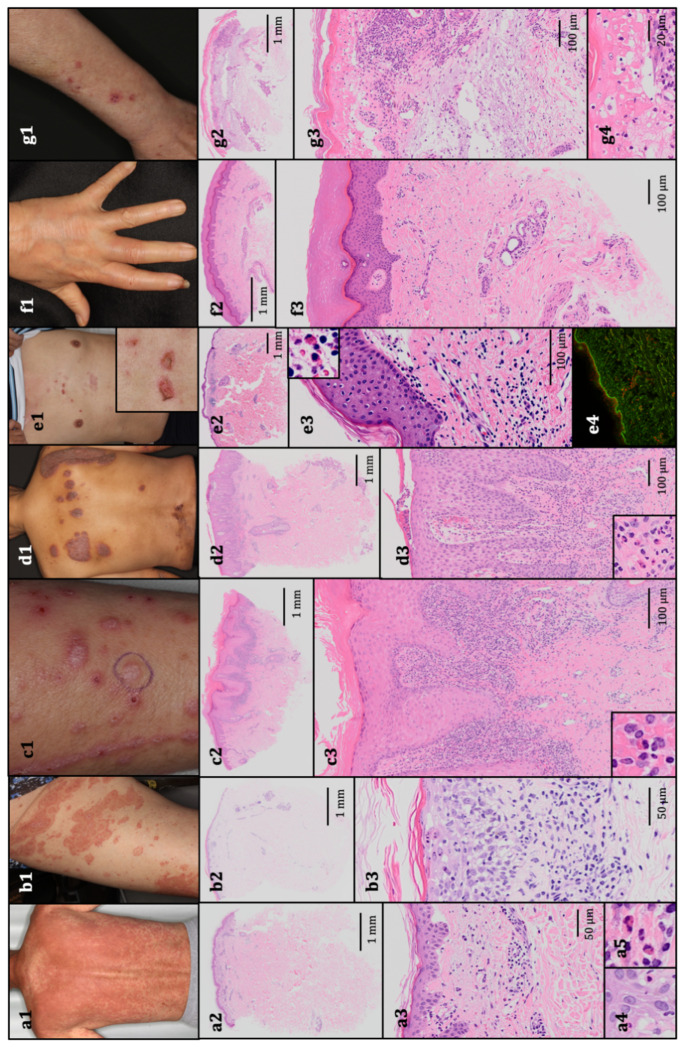
The representative clinical presentations and histopathological features. (**a1**) Maculopapular rash, grade 3. The patient received pembrolizumab for stage IV non-squamous non-small-cell lung cancer. Histopathology revealed vacuolar degeneration at the dermal–epidermal junction, perivascular infiltration of lymphocytes and eosinophils, and necrotic keratinocytes (hematoxylin and eosin (HE); (**a2**) 10×, (**a3**) 200×, and (**a4**,**a5**) 400× original magnification). (**b1**). Erythema multiforme, grade 2. The patient received nivolumab for stage IV squamous non-small-cell lung cancer. Histopathology revealed vacuolar degeneration at the dermal–epidermal junction and perivascular infiltration of lymphocytes and eosinophils, histopathologically resembling maculopapular rash (HE; (**b2**) 10× and (**b3**) 200× original magnification). (**c1**). Lichenoid reaction, grade 1. The patient received nivolumab for metastatic melanoma. Histopathology revealed the lichenoid infiltration of lymphocytes and a few eosinophils, and the epidermis exhibited acanthosis, a thickened granular layer, orthokeratotic hyperkeratosis, and spongiosis (HE; (**c2**) 10× and (**c3**) 100× original magnification). (**d1**) Psoriasiform reaction, grade 2. The patient received atezolizumab for stage IV non-squamous non-small-cell lung cancer. Histopathology revealed epidermal hyperkeratotic parakeratosis and acanthosis without a granular layer, elongation of rete ridges and dermal papilla, mild vacuolar degeneration at the dermal–epidermal junction, and perivascular infiltration of lymphocytes, eosinophils, and neutrophils in the upper dermis (HE; (**d2**) 10× and (**d3**) 100 × original magnification). (**e1**). Bullous pemphigoid, grade 1. The patient received pembrolizumab for metastatic melanoma. Histopathology revealed subepidermal bulla with eosinophils and perivascular infiltration of lymphocytes and eosinophils (HE; (**e2**) 10× and (**e3**) 100× original magnification). (**e4**) A direct immunofluorescence test revealed the linear deposition of IgG. (**f1**). Scleroderma-like reaction, grade 1. The patient received nivolumab for stage IV gastric adenocarcinoma. Histopathology revealed an increased amount of thick collagen fibers that packed sweat glands (HE; (**f2**) 10× and (**f3**) 100× original magnification). (**g1**). Stevens–Johnson syndrome, grade 3. The patient received nivolumab for stage IV non-squamous non-small-cell lung cancer. Histopathology revealed epidermal necrosis with numerous necrotic keratinocytes, acantholytic bullae, infiltration of lymphocytes and eosinophils, and parakeratotic hyperkeratosis (HE; (**g2**) 10×, (**g3**) 100×, and (**g4**) 400× original magnification).

Diagnoses were rendered via clinicopathological correlation. Of the 51 cutaneous irAEs identified via skin biopsy, the most common rash type was maculopapular rash (38/51, 74.5%), namely scattered edematous macules and/or red papules. The diagnosis of maculopapular rash was also rendered in cases with scattered papules, even if there was a possibility of a fused target lesion forming an erythematous plaque. The irAE of maculopapular rash was similar to typical exanthematous drug eruptions secondary to antibiotics, nonsteroidal anti-inflammatory drugs, and other treatments. The distribution of maculopapular rash was trunk-predominant (*n* = 16), extremity-predominant (*n* = 9), trunk alone (*n* = 7), or extremities alone (*n* = 6). Histopathologically, vacuolar degeneration at the dermal–epidermal junction and perivascular infiltration of lymphocytes were observed. Eosinophilic infiltration was not evident in some cases (*n* = 8). Other rash types included erythema multiforme (2/51, 3.9%), lichenoid reaction (3/51, 5.9%), psoriasiform reaction (3/51, 5.9%), bullous pemphigoid (3/51, 5.9%), scleroderma-like reaction (1/51, 2.0%), and SJS (1/51, 2.0%). Histopathologically, erythema multiforme shared similar features to maculopapular rashes (vacuolar degeneration at the dermal–epidermal junction, perivascular infiltration of lymphocytes, and infiltration of eosinophils). Lichenoid reactions had lichen planus-like clinical features, including pink-to-violaceous scaly papules. Oral ulcers and leukoplakia were observed in one case, and nail dystrophy was not evident in our patients. Psoriasiform reactions were similar to typical psoriasis vulgaris, including plaque psoriasis with well-defined, reddish-pink papules and plaques with silvery scales. Histopathologically, we observed epidermal hyperkeratotic parakeratosis and acanthosis without a granular layer, elongation of rete ridges, and dermal papillae. Perivascular infiltration of lymphocytes, eosinophils, and neutrophils at the upper dermis was also observed. In cases of bullous pemphigoid, eroded bullae with erythematous macules appeared together with histopathological subepidermal bullae and eosinophilic infiltrate. An additional direct immunofluorescence test was performed, and linear deposits of IgG and C3 were identified. Overall, cutaneous irAEs shared clinical and histopathological features with classical inflammatory eruptions from HE specimens. Clinical presentations and histopathological features are summarized in Table 2, and examples of typical findings are presented in Figure 1.

Of the 51 rashes, 28 (54.9%) were grade 1, 17 (33.3%) were grade 2, and 6 (11.8%) were grade 3, and no grade 4 rash was observed. The grade 3 rashes included maculopapular rashes and SJS. No patients died of cutaneous irAEs.

### 3.3. Rash Characteristics

Table 3 summarizes the patient demographics, associated ICIs, rash characteristics, and other irAEs according to the rash type. Maculopapular rash was the most common inflammatory eruption in each ICI class (anti-PD-1 antibody, 26/37; anti-PD-L1 antibody, 5/6; anti-CTLA-4 antibody, 2/2; and combined anti-PD-1 and anti-CTLA-4 therapy, 5/6). However, no obvious correlation was found between ICI classes and rash type. Maculopapular rash was the most common type in patients with lung cancer and melanoma (*n* = 14 and *n* = 10, respectively). Overall, no specific trends were observed between tumor types and rash types.

The duration from the start of ICI treatment to cutaneous reaction varied according to rash type. Maculopapular rash, erythema multiforme, and SJS had a short median latency, ranging from 11.0–94.5 days. Conversely, lichenoid reaction, psoriasiform reaction, bullous pemphigoid, and scleroderma-like eruption had a longer median latency, ranging from 140.0–231.0 days. The longest latency of 509 days (32 cycles of nivolumab completed) was noted in a patient with maculopapular rash. The shortest latency of zero days (six hours after administering pembrolizumab) was also noted in a patient with maculopapular rash. Pruritus was observed in 29 patients (56.9%), and there were one case of maculopapular rash and one case of lichenoid reaction with mucosal lesions other than SJS. The blood eosinophil percentage was elevated in patients with maculopapular rash, erythema multiforme, lichenoid reaction, psoriasiform reaction, bullous pemphigoid, and SJS (the range of median percentage, 4.8–19.8%). By contrast, the blood eosinophil count was not significantly elevated in patients with scleroderma-like eruption.

Other irAEs, including adrenalitis, colitis, hepatitis, cholangitis, diabetes mellitus, hypophysitis, myocarditis, pneumonitis, thyroiditis, and parotitis, were found in patients with maculopapular rash (*n* = 9) and lichenoid reaction (*n* = 2).

### 3.4. Treatment and Impact on Immunotherapy

The ICI classes, treatments for rash, impact on immunotherapy, and response to dermatologic therapy or interruption of immunotherapy according to the grade of cutaneous irAE are summarized in Table 4. The combined use of anti-PD-1 and anti-CTLA-4 antibodies tended to be associated with a higher grade of rash. Grade 3 rash was observed in patients receiving anti-PD-1 therapy (*n* = 4) and combined anti-PD-1 and anti-CTLA-4 therapy (*n* = 2).

In our study, 23 of 51 (45.1%) rashes were treated with systemic steroids, namely prednisolone at doses of <0.4 mg/kg (7/23, 30.4%), 0.4–1.0 mg/kg (9/23, 39.1%), and >1.0 mg/kg (7/23, 30.4%). In three cases, steroid pulse therapy (intravenous methylprednisolone 1000 mg/day for 3 consecutive days) was used. Two of three patients were receiving combination therapy with anti-PD-1 and anti-CTLA-4 antibodies, and the other patient was treated with anti-PD-1 antibody monotherapy. All of three rashes were maculopapular rashes. In three cases of psoriasiform reaction (*n* = 3), topical active vitamin D was added to topical steroid and systemic antihistamine. Intravenous immunoglobulin at a dose of 400 mg/kg/day for 5 consecutive days was administered for the patient with SJS.

Interruption of ICI therapy occurred in multiple rash types because of the eruption severity. In total, 7 of 51 cases (13.7%) resulted in temporary interruption and 7 (13.7%) resulted in permanent discontinuation due to cutaneous irAEs. Of seven cases with permanent discontinuation due to cutaneous irAEs, three cases were grade 2 rash and four cases were grade 3. Three patients (5.9%) with temporary interruption and nine patients (17.6%) with permanent discontinuation were interrupted of ICI therapy due to the progression of the disease or extracutaneous irAEs (e.g., adrenalitis, colitis, hepatitis, or pneumonitis) occurring at the same time. Immunotherapy was continued without interruption in 25 of 51 patients (49.0%).

In total, 50 of 51 (98.0%) rashes (including those that were exacerbated on subsequent ICI administration) improved with appropriate dermatologic therapy, interruption of immunotherapy, or both. Only one rash with bullous pemphigoid (rash grade 1) did not respond to dermatologic treatment and the new growth of bullae continued mildly. However, the symptoms were self-limiting, and we continued ICI therapy with topical steroid and systemic antihistamines.

## 4. Discussion

In this study, we summarized data on 51 cases of irAEs. Several retrospective studies and reviews have summarized the clinical and histopathological features of cutaneous irAEs, but none of these studies examined Asians. Our study is the first report of a detailed examination of the clinical and histopathological findings of cutaneous irAEs in a relatively large cohort experienced in Japan. The most common cutaneous irAEs was maculopapular rash, but ICIs caused virtually every type of skin rash, including erythema multiforme, lichenoid reaction, psoriasiform reaction, autoimmune diseases such as scleroderma-like reaction and bullous pemphigoid, and severe drug eruptions such as SJS. Interestingly, cutaneous irAEs shared clinical and histopathological features with classical inflammatory eruptions. Although not observed in our study, there have been reports of sarcoidosis [16], Grover’s disease [17,18,19], granuloma annulare [20], dermatomyositis [21,22], Sjögren’s syndrome [23], pityriasis rubra pilaris [24], or acute generalized exanthematous pustulosis [25,26] induced by ICIs. Several reports [27,28,29] suggested that in superficial perivascular dermatitis induced by ICIs, there were increased numbers of CD4^+^ lymphocytes compared with CD8^+^ lymphocytes, as well as regulatory T cells. However, the application of differentiation from conventional drug eruptions is controversial.

The incidence of ICI-induced inflammatory eruptions is the highest among irAEs, with anti-PD-1 and anti-CTLA-4 antibodies causing events in 20–30% and nearly 50% of patients, respectively [8,9,30]. However, most events are self-limiting (grades 1–2). In this study, 45 of 51 (88.2%) rashes were grades 1–2, covering less than 30% of the BSA, and grade 3 or higher rashes accounted for 11.8% of all rashes. There was a trend toward higher-grade rash compared to previous reports [5,8,9], probably because patients in this cohort were evaluated only when they were referred to our dermatology department for expert advice and skin biopsy. In this study, two cases of grade 3 cutaneous irAEs were attributable to combination treatment with anti-PD-1 and anti-CTLA-4 antibodies, and the combination therapy tended to cause more severe inflammatory eruptions than anti-PD-1 or anti-CTLA-4 antibody monotherapy. Thus, combination therapy may increase the grade of rash and the frequency of cutaneous irAE.

Cutaneous irAEs may occur first among all irAEs [8,9,30], and the onset of rash varies by rash type. In our study, maculopapular rash, erythema multiforme, and SJS had a relatively early onset, with most of them occurring within 3 months after the first ICI dose. Conversely, lichenoid reactions and autoimmune diseases such as bullous pemphigoid and scleroderma-like reactions occurred relatively late, emerging 4–8 months after treatment initiation. Previous case reports and reviews indicated that maculopapular rash often develops 3–6 weeks after the first use of ICIs [8,9,31,32], lichenoid reactions occur after 6–12 weeks [6,8,9,32,33,34], and bullous pemphigoid appears after approximately 14 weeks [6,34,35]. Although several reports suggested a relatively early onset of psoriasiform reactions [36,37,38], psoriasiform reactions occurred more than 26 weeks after treatment initiation in two of three patients. Excluding the maculopapular type, only a few cases were included in each rash type, and further accumulation of cases is required.

Cutaneous irAEs were treated according to the rash grade. More than half of grade 2 rashes were treated with systemic steroids, and patients with grade 3 or higher rashes were often treated with high-dose prednisolone (≥1 mg/kg). In the case of psoriasiform reaction, topical active vitamin D was added to the treatment. However, there was no apparent difference in the choice of treatment by rash type. Among patients with grade 1 rash, ICIs were permanently discontinued in some patients (*n* = 3) due to extracutaneous irAEs or patients’ preference. Meanwhile, ICIs can be continued and the skin rash will improve without exacerbation following dermatologic treatments (e.g., topical steroids, oral antihistamines, and moisturizers) in most cases. Recently, cutaneous irAEs were recognized to be amenable to topical treatment, without the need for medication dose reduction or discontinuation [5,39]. In addition, ICIs can be resumed after tentative interruption. In our cohort, ICI therapy was permanently discontinued due to cutaneous irAEs in only 7 of 51 cases (13.7%). Cutaneous irAEs were not dose-dependent [9]. Notably, it is recommended that ICI resumption be considered in consultation with dermatologists after resolution of skin toxicity, even in severe cases [40]. In the current study, most patients were treated before the establishment of the management of cutaneous irAE; therefore, they were treated with systemic steroids.

This study had several limitations. First, this was a retrospective, single-institutional study. Patients were evaluated only when they were referred to our dermatology department for expert advice and skin biopsy. Mild cases in which a skin biopsy was unnecessary were not evaluated. Thus, our cohort may represent more severe inflammatory eruptions at our institution. Additionally, there may be a potential bias in the rash types. Only one or two cases of minor skin rash were included. Further accumulation of cases is needed to compare the timing of onset and grade of rash.

## 5. Conclusions

We summarized the clinical characteristics and histopathological findings of 51 cases of biopsy-proven cutaneous irAEs induced by ICIs. Although various inflammatory eruptions occur in the treatment of ICIs, the inflammatory eruptions induced by ICIs share similar clinical and histopathological features with classical inflammatory eruptions. Differing from irAEs in other organs, some of cutaneous irAEs may be equivalent to conventional drug eruptions. Further research is warranted.

## Figures and Tables

**Table 1:** Demographics, underlying malignancy, and immunotherapy of the trial participants.

Parameter	Value (%)
Age, years	
Range (mean ± SD)	39–85 (65.5 ± 10.8)
Sex	
Male	30 (58.8)
Female	21 (41.2)
Underlying malignancy	
Cutaneous	19 (37.3)
Melanoma	12 (23.5)
Merkel cell carcinoma	1 (2.0)
SCC of head and neck	6 (11.8)
Lung	18 (35.3)
Sq NSCLC	7 (13.7)
Non-Sq NSCLC	10 (19.6)
SCLC	1 (2.0)
Gastrointestinal	5 (9.8)
Esophageal SCC	3 (5.9)
Gastric adenocarcinoma	2 (3.9)
Genitourinary	6 (11.8)
Renal cell carcinoma	5 (9.8)
Urothelial carcinoma	1 (2.0)
Lymphoma	3 (5.9)
Immune checkpoint inhibitors	
Nivolumab	24 (47.1)
Pembrolizumab	13 (25.5)
Ipilimumab	2 (3.9)
Nivolumab + ipilimumab	6 (11.8)
Atezolizumab	4 (7.8)
Avelumab	2 (3.9)
Total	51 (100.0)

SCC, squamous cell carcinoma; Sq NSCLC, squamous non-small cell lung cancer; Non-Sq NSCLC, non-squamous non-small cell lung cancer.

**Table 2:** Clinical and histopathological findings and the grade of inflammatory eruption.

Rash Type (*n*)	Clinical Presentation (*n*)	Corresponding Histopathological Features (*n*)	Grade, *n*			
			1	2	3	4
Maculopapular (38)	Scattered edematous macules and/or red papules trunk-predominant (16), extremity-predominant (9), trunk alone (7), extremities alone (6)	Vacuolar degeneration at the DEJ (38), perivascular lymphocytic infiltration (38), eosinophilic infiltration (30), epidermal spongiosis (16), necrotic keratinocytes (15), small abscess in the epidermis (2)	23	10	5	0
EM (2)	Erythematous macules with target lesion or iris formation, scattered on the trunk and proximal extremities (2)	Vacuolar degeneration at the DEJ (2), perivascular lymphocytic infiltration (2), eosinophilic infiltration (2), epidermal spongiosis (1), necrotic keratinocytes (1)	0	2	0	0
Lichenoid (3)	Pink-to-violaceous papules and plaques with scales, predominantly on the extremities (3), oral ulcer and leukoplakia (1)	Dense lymphocytic infiltration at the DEJ (lichenoid infiltration) (3), infiltration of a few eosinophils (3), necrotic keratinocytes (3), acanthosis (2), thickened granular layer (2), orthohyperkeratosis (2), and epidermal spongiosis (2)	2	1	0	0
Psoriasiform (3)	Plaque psoriasis on the trunk and extremities, with no pustulosis, scalp lesions, or arthritis (3)	Parakeratosis, acanthosis, diminished granular layer, elongated rete ridges, intraepidermal bullae containing neutrophils, mild vacuolar degeneration at the DEJ, and perivascular infiltration of lymphocytes, eosinophils, and neutrophils in the upper dermis (3)	2	1	0	0
BP (3)	Eroded bullae with erythematous macules on the chest and abdomen (3)	Subepidermal bulla containing eosinophils, perivascular infiltration of lymphocytes and eosinophils (3), linear deposition of IgG and C3 at the DEJ on DIF (3)	1	2	0	0
Scleroderma-like (1)	Skin sclerosis of the fingers (1)	Increased thick collagen bundles packing sweat glands (1)	0	1	0	0
SJS (1)	Erythematous macules scattered on the trunk and proximal extremities, mucosal ulcerations, the Nikolsky sign (1)	Epidermal necrosis with numerous necrotic keratinocytes, acantholytic bullae, infiltration of lymphocytes and eosinophils, and parakeratotic hyperkeratosis (1)	0	0	1	0
Total			28	17	6	0

EM, erythema multiforme; BP, bullous pemphigoid; DEJ, dermal–epidermal junction; DIF, direct immunofluorescence; C3, complement 3; SJS, Stevens–Johnson syndrome.

**Table 3:** Summary of patient demographics, associated immunotherapy class, rash characteristics, and other irAEs.

Rash Type (*n*)	Demographics		Immunotherapy Class, *n*				Rash Characteristics				Other irAEs, *n*
	Age, years, Mean	Sex, Male, *n*	Anti-PD-1	Anti-PD-L1	Anti-CTLA-4	Combined Anti-PD-1 and Anti-CTLA-4	Latency to irAEs, Days, Median (Range)	Pruritus, *n*	Mucosal Lesion, *n*	Median Blood eos, %	
Maculopapular (38)	64.2	19	26	5	2	5	24.5 (0–509)	22	2	5.1	9
EM (2)	74.0	2	2	0	0	0	94.5 (49–140)	1	0	5.2	0
Licehnoid (3)	67.0	1	3	0	0	0	169.0 (120–255)	1	1	5.2	2
Psoriasiform (3)	65.0	3	1	1	0	1	185.0 (4–344)	2	0	10.9	0
BP (3)	71.2	3	3	0	0	0	231.0 (139–365)	2	1	4.8	0
Sclerodermoid (1)	73.0	1	1	0	0	0	140.0	0	0	0.9	0
SJS (1)	69.0	1	1	0	0	0	11.0	1	1	19.8	0
Total (51)	65.5	30	37	6	2	6	50 (0–509)	29	5	5.3	11

Abbreviation: irAEs, immune-related adverse events; PD-1, programmed cell death 1; PD-L1, programmed cell death ligand 1; CTLA-4, cytotoxic T-lymphocyte-associated protein 4; eos, eosinophil; EM, erythema multiforme; BP, bullous pemphigoid; SJS, Stevens–Johnson syndrome.

**Table 4:** ICI therapy class, rash treatment, impact on immunotherapy, and therapeutic response according to the rash grade.

Rash Grade (*n*)	Immunotherapy Class, *n*				Rash Treatment ^†^, *n*				Impact on Immunotherapy, *n*					Rash Improved, *n*, yes/no
	Anti-PD-1	Anti-PD-L1	Anti-CTLA-4	Combined Anti-PD-1 and Anti-CTLA-4	1	2	3	4	None	Temporarily Interrupted		Permanently Discontinued		
										DCI	DOC	DCI	DOC	
1 (28)	21	4	2	2	23	5	0	0	22	2	1	0	3	27/1
2 (17)	12	2	0	2	5	2	8	2	3	4	1	3	6	17/0
3 (6)	4	0	0	2	0	0	1	5	0	1	1	4	0	6/0
4 (0)	0	0	0	0	0	0	0	0	0	0	0	0	0	0/0
Total (51)	37	6	2	6	28	7	9	7	25	7	3	7	9	50/1

^†^ The rash treatment was as follows: 1, only topical steroids or systemic antihistamines; 2, systemic steroids (<0.4 mg/kg prednisolone) and/or topical steroids; 3, systemic steroids (0.4–1.0 mg/kg prednisolone) and/or topical steroids; 4, systemic steroids (>1.0 mg/kg prednisolone) and/or topical steroids. ICI, Immune checkpoint inhibitor; PD-1, programmed cell death 1; PD-L1, programmed cell death ligand 1; CTLA-4, cytotoxic T-lymphocyte-associated protein 4; DCI, due to cutaneous immune-related adverse events; DOC, due to other causes.

## Data Availability

The data presented in this study are available on request from the corresponding author. The data are not publicly available due to privacy restrictions.
